# Development of Hourly Resolution Air Temperature Across Titicaca Lake on Auxiliary ERA5 Variables and Machine Learning-Based Gap-Filling

**DOI:** 10.3390/s25237165

**Published:** 2025-11-24

**Authors:** Jimmy W. Sirpa-Poma, Juan Calle, Elvis Uscamayta-Ferrano, Jorge Molina-Carpio, Frederic Satgé, Osmar Cuentas Toledo, Ricardo Duran, Paula Pacheco Mollinedo, Riaz Hussain, Ramiro Pillco-Zolá

**Affiliations:** 1 Instituto de Hidráulica e Hidrología, Universidad Mayor de San Andrés, La Paz, Bolivia; 2 Instituto de Investigaciones Físicas, Universidad Mayor de San Andrés, La Paz, Bolivia; juanmarcoscalle@chacaltaya.edu.bo; 3 Graduate Program in Applied Geosciences and Geodynamics (PPGGAG), Institute of Geosciences, University of Brasília (UnB), Brasília 70910-900, Brazil; 241130260@aluno.unb.br; 4 ESPACE-DEV, University Montpellier, IRD, Univ Antilles, Univ Guyane, University Réunion, 34093 Montpellier, France; frederic.satge@ird.fr (F.S.); paula.pacheco-mollinedo@ird.fr (P.P.M.); 5 Grupo de Investigación de Ingeniería Civil, Escuela Profesional de Ingeniería Civil, Universidad Nacional de Moquegua, Moquegua 180101, Peru; ocuentast@unam.edu.pe; 6 Ministerio de Desarrollo Agrario y Riego del Perú, Lima 150114, Peru; proyectos75@sierraazul.gob.pe; 7 Department of Agronomy, The University of Haripur, Haripur 22621, Pakistan; riazkhosa11092@gmail.com

**Keywords:** Titicaca Lake, air temperature, quality control, machine learning, gap-filling, data consistency

## Abstract

This article presents an innovative procedure that combines advanced quality control (QC) methods with machine learning (ML) techniques to produce reliable, continuous, high-resolution meteorological data. The approach was applied to hourly air temperature records from six automatic weather stations located around Lake Titicaca in the Altiplano region of South America. The raw dataset contained time gaps, inconsistencies, and outliers. To address these, the QC stage employed Interquartile Range, Biweight, and Local Outlier Factor (LOF) statistics, resulting in a clean dataset. Two gap-filling methods were implemented: a spatial approach using time series from nearby stations and a temporal approach based on each station’s time series and selected variables from the ERA5-Land reanalysis. Several ML models were also employed in this process: Random Forest (RF), Support Vector Machine (SVM), Stacking (STACK), and AdaBoost (ADA). Model performance was evaluated on a validation subset (30% of station data). The RF model achieved the best results, with R^2^ values up to 0.9 and Root Mean Square Error (RMSE) below 1.5 °C. The spatial approach performed best when stations were strongly correlated, while the temporal approach was more suitable for locations with low inter-station correlation and high local variability. Overall, the procedure substantially improved data reliability and completeness, and it can be extended to other meteorological variables.

## 1. Introduction

Temporal high-resolution climate data introduces significant challenges to maintaining the consistency and reliability of climate records [1,2]. Consequently, rigorous data quality control (QC) procedures are indispensable, both to safeguard data integrity and to ensure the reliability of downstream applications such as numerical weather prediction, hydrometeorological early warning systems, drought monitoring, and climate-informed decision-making [3,4,5,6,7]. While a wide range of QC methodologies have been developed, varying in scope and complexity, their applicability is not uniform across climatic regions or dataset structures. Such limitations are particularly acute in regions like the Central Andes, where the spatial density of Automatic Weather Stations (AWS) remains limited.

Temporal consistency tests are applied to individual time series or, where data availability and spatial representativeness permit, across neighboring series [8,9,10,11]. While basic statistical measures (e.g., histograms, standard deviation, autocorrelation) describe data variability and aid in outlier detection, Quality Control (QC) procedures specifically identify anomalies arising from sensor malfunctions, transmission errors, or even valid extreme climatic events [10,12,13]. Station-specific QC is therefore critical in remote regions, where a lack of neighboring stations precludes spatial consistency checks [14].

Commonly used outlier detection algorithms—such as Interquartile Range (IQR), Biweight, and Local Outlier Factor (LOF)—are effective for non-normally distributed data [15]. These are supplemented by additional consistency tests, including range checks, persistence analysis, and step-change detection [16,17,18,19,20].

Quality-controlled time series require gap-filling for continuity [19]. After verifying that inconsistencies have been properly addressed, machine learning (ML) algorithms are increasingly used for this task. ML algorithms excel at capturing the nonlinear relationships inherent in complex climatic systems [21,22]. By integrating these physically relevant predictors, the models significantly enhance the representativeness and statistical reliability of the gap-filled dataset, surpassing the capabilities of traditional interpolation methods [23]. This robust output is crucial for subsequent hydro-climatic analysis and extreme event detection.

Since 2016, a network of AWS has been deployed along the shores of Lake Titicaca in the Altiplano of South America to provide continuous meteorological records, including hourly average air temperature ($T_{air}$). Ensuring the robustness and temporal consistency of these records necessitates the implementation of a well-structured workflow and robust procedures. Recent studies show that global climate change is affecting the Altiplano, particularly through decreased seasonal precipitation and increased thermal variability, which threaten the water balance of the Lake Titicaca basin [24]. These trends highlight the critical need for robust quality-controlled climatic data.

This study primarily aims to generate consistent time-series datasets of hourly $T_{air}$ from continuous automatic data-logger records, while also establishing a methodology for gap-filling in $T_{air}$ observations around Lake Titicaca. The approach integrates temporal and spatial analyses, supported by auxiliary ERA5-Land reanalysis datasets and machine learning (ML) models. The workflow includes the following: (i) raw data unification from all automatic weather stations, recorded at different sampling intervals; (ii) the application of three robust quality-control (QC) procedures to enhance data quality and reliability; (iii) consistency tests (CT); (iv) the implementation of four ML techniques for synthetic $T_{air}$ dataset generation, followed by gap-filling to ensure complete consistency.

## 2. Study Area and Automatic Monitoring

Lake Titicaca, shared by Peru and Bolivia, is situated in the northern Altiplano (14–17° S, 71–68° W). It has a 57,000 km^2^ watershed averaging 4000 m.a.s.l. in elevation [25,26]. With a surface area of 8500 km^2^, it is South America’s largest lake and significantly modulates the regional climate due to its size and altitude.

The regional semi-arid climate features a short rainy season (December–March) and a prolonged dry season (April–November), with a mean annual air temperature of ~7 °C and diurnal shore fluctuations from −1.5 to 20 °C [27]. The lake’s surface water temperature averages 4 °C higher than the daytime air temperature [25]. Northwest–southeast winds dominate, influencing evaporation, water mixing, and the transport of nutrients and pollutants [28,29,30].

These distinctive climatic features result from the complex interaction between atmospheric dynamics, regional geography, and the lake itself, creating a microclimate.

## 3. Data and Methods

### 3.1. Data

#### 3.1.1. $T_{air}$

**Figure 1:**
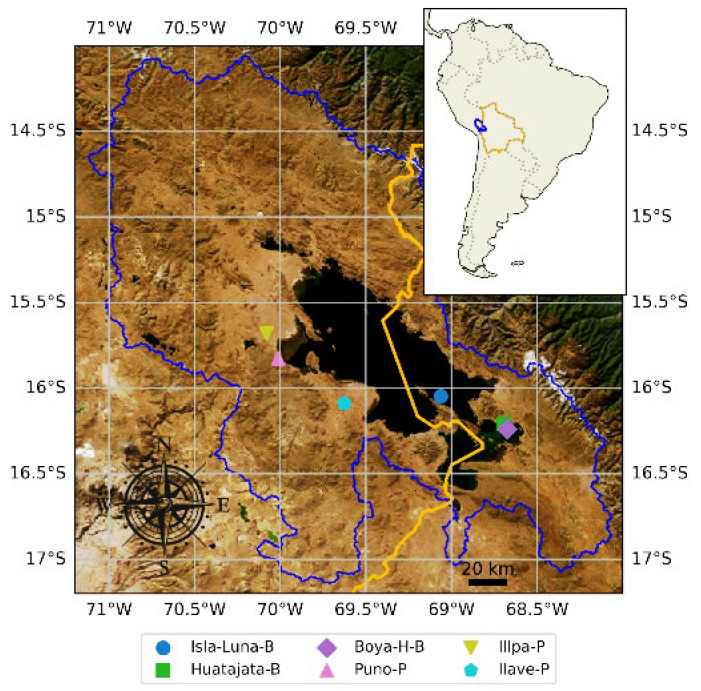
Titicaca Lake basin with automatic climate monitoring stations. The orange line indicates the Bolivian border, and the blue line outlines the Titicaca basin.

A high-resolution automatic weather monitoring network was recently established around Lake Titicaca, consisting of six stations installed between 2016 and 2022 (Table 1, Figure 1). On the Bolivian side, three stations—Isla-Luna-B, the lakeshore station Huatajata-B, and the lacustrine station Boya-HidroMet-B—are operated by the Instituto de Hidráulica e Hidrología and the Observatorio Permanente del Lago Titicaca (La Paz). Three stations on the Peruvian side—Puno-P, Illpa-P, and Ilave-Peru—are operated by SENAMHI-Peru. The spatial distribution of these stations is shown in Figure 1. With the exception of the Huatajata-B and Boya-HidroMet-B stations, which are proximal (4.5 km apart), inter-station distances range from 80 to 250 km.

The monitoring network includes stations in lacustrine, littoral, and inland environments. Significant differences in mean hourly air temperature ($T_{air}$) were observed between these stations, attributable to the distinct thermal properties of their surroundings. Inland stations (e.g., Illpa-P, Ilave-P) exhibited rapid heating and cooling due to the relatively high thermal conductivity of land surfaces. In contrast, the lacustrine station (Boya-H-B) demonstrated attenuated temperature fluctuations, consistent with the high heat capacity of water. A shoreline station (Isla-Luna-B) displayed intermediate thermal behavior.

The raw data contained errors and inconsistencies, such as duplicate records and heterogeneous sampling intervals (5, 15, 60 min), resulting from differing data acquisition protocols between the operating institutions. To ensure consistency, all records were resampled to a common hourly time step. Custom Python scripts, developed using version 3.10, were created to standardize the database by generating regular hourly time series. The resulting homogeneous dataset spans from 13 September 2019, 00:00 to 7 April 2022, 13:00 for four stations, and from 1 May 2020, 00:00 to 7 April 2022, 13:00 for the remaining two.

#### 3.1.2. ERAA5-Land Hourly Dataset

Missing air temperature ($T_{air}$) data were reconstructed using ECMWF ERA5-Land reanalysis data as a secondary source. This dataset provides hourly atmospheric variables at an 11 km spatial resolution and is widely employed for quality control and climatological analysis [39,40]. The ERA5-Land variables utilized included air temperature ($T_{air}$), precipitation (PP), evaporation (Ew), relative humidity (Rh), solar radiation (Gr), wind speed (Ws), and potential evaporation (Pe). While ERA5-Land may not fully resolve localized diurnal cycles or high-frequency variability, its integration with quality-controlled in situ observations improves $T_{air}$ estimates.

To address spatial and temporal gaps, we applied traditional spatial interpolation techniques, including linear correlation regression (LR), and tested their findings using machine learning (ML) models (RF, SVM, STACK, and ADA.). These models evaluated the correlation between $T_{air}$ time series across stations and used auxiliary variables from the ERA5-Land reanalysis dataset.

Each model was trained and validated using both Raw and Clean datasets. This process included separate stages of calibration, testing, and final validation to ensure robustness.

### 3.2. Methods

**Figure 2:**
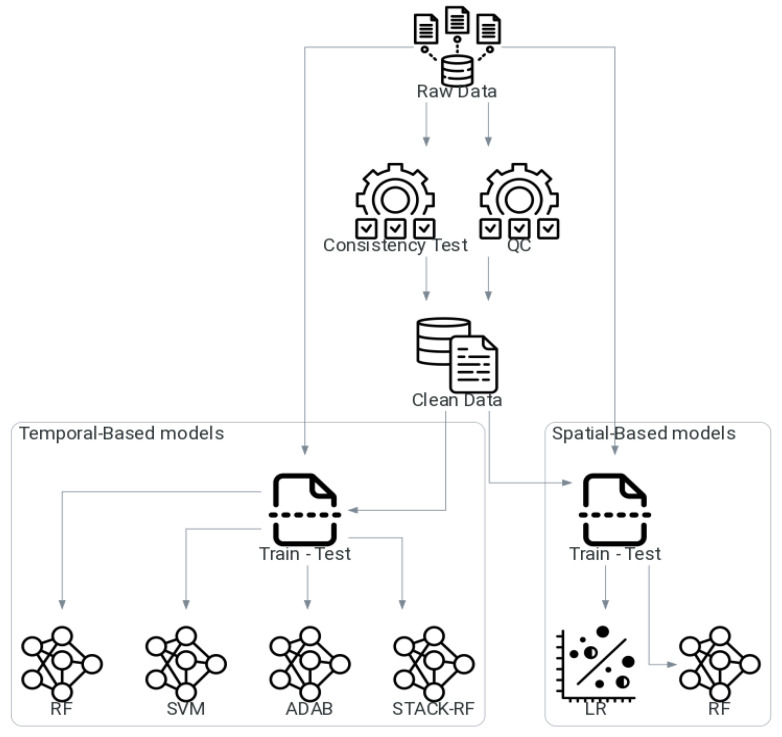
Air temperature modeling framework flowchart.

We employed a multi-step methodology to generate a consistent, homogenous hourly $T_{air}$ dataset for the Lake Titicaca region, as summarized in Figure 2. The flowchart outlines the sequential stages of data processing, quality control, and gap-filling performed prior to model evaluation and validation.

#### 3.2.1. Quality Control of $T_{air}$

##### Robust QC Analysis

Outliers were detected using three non-parametric statistical techniques: The Interquartile Range (IQR) [9,41], Biweight, and Local Outlier Factor (LOF) methods [42,43,44]. These techniques were selected for their ability to detect outliers without requiring assumptions about the data distribution. To maximize reliability and minimize false positives, a conservative approach was adopted: a data point was classified as an outlier only upon consensus from all three methods. Global outliers were identified through an analysis of the complete temporal record for each station [45,46,47]. A consistency test (CT) with predefined magnitude thresholds was also applied to identify persistent faults indicative of sensor malfunction.

Based on the quality control (QC) and CT results, two dataset versions were generated: a complete raw data version retaining all original measurements, and a clean data version with all identified outliers and faulty readings removed.

##### Interquartile Range (IQR)

This approach efficiently mitigates the influence of distribution tails while preserving information about extreme events, ensuring that these phenomena do not disproportionately affect statistical estimates. It is widely used in climate data quality control because the IQR is robust against outliers. While some identified outliers may represent erroneous data that can be removed or treated as missing, others are physically meaningful [48]. This balance allows retention of important extreme-event information while reducing the likelihood of bias. This method is particularly suitable for environmental data, which often exhibit non-normal and skewed distribution [49].

In this study, extreme values were trimmed using predefined thresholds: Pout75 = $q_{0.75}+k\cdot IQR$, Pout25 = $q_{0.25}-k\cdot IQR$, where $q_{0.75}$ and $q_{0.25}$ are the third and first quartiles, and IQR is the interquartile range. Following Tukey [50], we set k=3 to identify extreme outliers.

##### Biweight Approach

This method employs a robust statistical approach, using the median and the median absolute deviation (MAD) to redefine traditional measures of central tendency and dispersion, reducing sensitivity to outliers [51]. Data points are weighted based on their proximity to the median, with weights symmetrically decreasing as a function of their Median Absolute Deviation (MAD)-scaled distance [52], minimizing the influence of extreme observations [52]. Potential outliers are classified by their biweight Z-scores; values exceeding 3, 4, or 5 standard deviations are assigned escalating flags to indicate increasing levels of suspicion. This stratified system facilitates the prioritization and review of anomalous data.

Observations were normalized using a scaling constant. Consistent with the established literature, which recommends values of *C* between 6 and 9 [51], a value of *C* = 7.5 was initially tested. Subsequent sensitivity analyses indicated that *C* = 7.1 yielded stable and statistically consistent results, and was therefore adopted for the final implementation [17].

##### Local Outlier Factor (LOF)

The Local Outlier Factor (LOF) algorithm is a non-parametric, unsupervised method for identifying local outliers. Its core principle is the detection of points with a local density significantly lower than that of their nearest neighbors, making it particularly effective for heterogeneous datasets with uneven cluster densities [44,53]. The algorithm operates by calculating a continuous anomaly score (the LOF) for each point based on the relative density of its k-nearest neighbors, typically using Euclidean or Manhattan distance [45]. This score provides a nuanced measure of deviation, allowing for the distinction between weak and strong outliers rather than applying a binary classification [54].

##### Consistency Test of $T_{air}$

To complement robust outlier identification, we performed a comprehensive consistency check on the hourly air temperature ($T_{air}$) data, implementing four specific tests as recommended by established methods [16,17,18,19,20]:

1. Range Test: It identifies and removes all $T_{air}$ values that fall outside the acceptable range based on long-term observational data. The thresholds for this test, defined as TLOW and THIGH, were derived from the DECADE database [55], which contains extensive historical $T_{air}$ records from a conventional weather network which has been monitoring the Lake Titicaca region since 1972.
2. Step Test: It checks the excess movement between consecutive hourly $T_{air}$ values at 1-, 2-, 3-, 6-, and 12-h intervals against limits of 4, 7, 9, 15, and 25 °C, respectively, to identify unrealistic rates of change.
3. Persistence Test (Flat Line Test): A series of identical values over a three-hour window—indicative of sensor malfunction—was flagged, a duration selected due to the high intra-diurnal $T_{air}$ variability at this latitude.

#### 3.2.2. Gap-Filling of $T_{air}$

##### Gap-Filling on Linear Correlation Regression

Meteorological data gap-filling commonly employs linear correlation regression (LR) or the normal ratio method, which require high correlation with a proxy station [56]. To select the most suitable stations for imputation, pairwise R^2^ and RMSE were first calculated using both the raw and cleaned datasets, allowing identification of the best candidate stations. Among the six stations considered, only Huatajata-B and Boya-H-B exhibited sufficient spatial correlation. The other stations, located over 50 km away, were excluded due to low correlations. However, proximity alone is insufficient; topographic and climatic heterogeneity must also be considered to ensure reliable imputations [57,58,59].

##### Gap-Filling on ML Models

We employed four machine learning (ML) algorithms known for their effectiveness in modeling complex, nonlinear climate relationships: Support Vector Machine (SVM), Random Forest (RF), AdaBoost (ADA), and Stacking (STACK) [23,60,61]. Environmental variables from the ERA5-Land reanalysis database (${T\prime }_{air}$, Pp, Ew, Ws, Wd, and Pe) were used. These variables are commonly accepted as an influential predictor in many hydrometeorological modeling activities [39,40,62,63].

- Support Vector Machine (SVM): Implemented with a polynomial kernel of degree 3, a penalty parameter *C* = 1, and a kernel coefficient of 1 [64].
- Random Forest (RF): This ensemble method aggregates predictions from multiple decision trees trained on random subsets of data and features, reducing variance and improving generalization. The model was configured with 90 and 100 trees, a maximum tree depth of 9, 6 samples to split an internal node, and a maximum of 3 features per split.
- AdaBoost (ADA): This iterative ensemble method combines weak learners by increasing the weight of misclassified instances, effectively reducing bias. The set-up used 80 estimators with a learning rate of 1.0, an approach proven effective for environmental regression tasks [65,66].
- Stacking (STACK): This advanced ensemble technique integrates the predictions of base models (SVM and RF in this study) using a meta-model to generate a final, refined prediction, leveraging the strengths of each constituent algorithm.

##### Model Training and Evaluation

To evaluate the gap-filling performance of the Machine Learning (ML) models and Linear Correlation Regression (LR), the dataset was partitioned into a 70% training set and a 30% independent (validation) test set, a standard practice in environmental modeling [67,68]. This hold-out validation scheme assessed the models’ ability to generalize to unseen data. The analysis was conducted under two data quality scenarios to determine the impact of preprocessing: (1) raw data: the original, unprocessed time series; (2) clean-data: the time series after quality control (Section 3.2.1).

Model performance was evaluated on the validation set using three statistical metrics: the coefficient of determination ($R^{2}$), which quantifies the proportion of variance in observed temperature values explained by the model; root mean squared error (RMSE), the typical magnitude of prediction error (°C), and bias, the average systematic over- or under-estimation of temperature values by the model. These indicators provide a general view of predictive accuracy, error magnitude, and systematic deviation, respectively. Their calculation followed standard formulations (Equations (1)–(3)), where $y_{i}$, ${\hat{y}}_{i}$, and ${\bar{y}}_{i}$ represent the observed, predicted, and mean values, respectively.

(1) $$R^{2}=1-\frac{\sum_{i=1}^{n}{\left(y_{i}-{\hat{y}}_{i}\right)}^{2}}{\sum_{i=1}^{n}{\left(y_{i}-{\bar{y}}_{i}\right)}^{2}}$$

(2) $$RMSE=\sqrt{\frac{1}{n}\sum_{i=1}^{n}{\left(y_{i}-{\hat{y}}_{i}\right)}^{2}}$$

(3) $$Bias=\frac{1}{n}\sum_{i=1}^{n}{\left({\hat{y}}_{i}-y_{i}\right)}^{2}$$

## 4. Results

The data in Table 2 is a very detailed description of every $T_{air}$ time series from our six different weather stations from the perspective of quality control. This table explains the coverage period of the data in each station, the percentage of data per station that was not captured, the total records of the captured raw data, the number of outliers detected, and the results after some basic tests of consistency. As seen in Table 2, the completeness of the raw data among the stations was varied, mainly based on the availability of data. Particularly, the highest percentages of missing data were seen in the Illpa-P and Boya-H-B stations, 5.87 and 7.73 percent, respectively. The rest of the stations, on the other hand, depicted little to no significant missing data, and the degree of data completeness relating to $T_{air}$ measurements was very high.

### 4.1. QC Analysis and Consistency Test

#### 4.1.1. Interquartile Range, Biweight, and LOF

To determine outliers in the hourly $T_{air}$, three means of quality control were put in place by looking at the six time series that were obtained from the weather stations; these were the Interquartile Range (IQR), Biweight, and the Local Outlier Factor (LOF). A data point was only identified as an outlier when it emerged as such in at least two of these methods. The determined outliers reflect $T_{air}$ values that did not align with the normal hourly distribution and were removed from the raw data. The summary of these outlier values can be seen in Table 2. The most outlying point’s decile was established in Isla-Luna-B (1.18%), and was 265 units removed from other $T_{air}$ data points. The smallest percentage was recorded in Huatajata-B (0.77%), which had 143 $T_{air}$ changes in values that were extracted out of the raw data.

#### 4.1.2. Consistency Test Values

A number of consistency tests were carried out to check the validity and conciseness of the $T_{air}$ time series data; in particular, aiming at faulty values, outliers, and time-sequential uniformity among all stations. These tests were as follows: 1: range test; 2: persistency test; 3: varying time lag step tests (1, 2, 3, 6, and 12 h). No violations were observed in the range test except in Illpa-P, which had 10 occurrences. The sensitivity of the step tests was, however, higher for detecting inconsistencies, especially in Illpa-P and Ilave-P. As an example, the 1-h step test marked out 5.97 and 2.12 percent of the values at these stations as discrepant, respectively. On the contrary, the results obtained for Boya-H-B and Isla-Luna-B demonstrated little inconsistency (0.005% and 0.013%, respectively), which supports the effectiveness of the 1-h step test in identifying sudden and possibly erroneous changes in temperature. After these quality control (QC) and consistency testing (CT) activities, we came up with a clean data $T_{air}$ set of data for each station. The statistical outliers were eliminated using robust techniques, i.e., Interquartile Range, Biweight, and Local Outlier Factor, to create the quality-controlled dataset (QC). ST-data—The additional data from the registered data points linked to the sharp and temporary changes in temperature detected in the course of the step tests were rejected. The processed datasets were performed more carefully, thus providing a more useful base from which later examination may be performed, such as data gap estimation and filling.

#### 4.1.3. Station-Specific Data Quality Observations

Table 2 shows that there is a large disparity between the quality of data presented by different AWS. These differences are due to variations in maintenance frequency, sensor types, or protocols used during the data recording process. For example, Huatajata-B and Isla-Luna-B exhibited no missing data and very low error rates, whereas Illpa-P showed the highest percentage of errors in the consistency tests (17.56%) with a data omission rate of 5.87%. The reduction in step test errors with extended time lags (2–12 h) was particularly notable at Illpa-P and Ilave-P, especially at shorter time lags. This may be attributed to a trend-breaking slip in temperature and possible intermittent sensor failures or a local microclimate anomaly. The low overlap rate between the outliers and the results of the consistency tests suggests that these two methods identify different types of data issues. However, it remains insufficiently explored how locally applicable these types of errors are.

### 4.2. Gap-Filling Performance

#### 4.2.1. Spatial Gap-Filling Approach of $T_{air}$

This technique for gap filling is the traditional one, wherein the proxy solutions are applied by using the linear correlation regression (LR) to relatively analyze the temporal series at neighboring stations [56]. Table 3 shows that the combination of pairwise comparisons among the six raw data series that exhibits the highest coefficient of determination ($R^{2}$) [69] is the correlation between these series, as the measured $R^{2}$ is equal to 0.96 and the corresponding RMSE averages out to 5.49 °C. These findings imply that a few $T_{air}$ time series may be gap filled with the simple LR model (especially those where $R^{2}$ is near to 0.90). Table 3 and Table 4 compare the spatial models that have a high correlation ($R^{2}$ up 0.96) with some of the AWS around Lake Titicaca, which makes the use of spatial interpolation ideal as the main method for gap filling. Although the linear regression model (LR-Spatial) provides satisfactory results, the Random Forest model (RF-Spatial) presents high values of $R^{2}$ and low RMSE in all stations, especially with preprocessed clean data. This means that the nonlinear or complicated temperate combinations will not be represented satisfactorily by linear regression, and more benefits are likely to be identified using machine learning models such as RF-Spatial [22].

**Figure 3:**
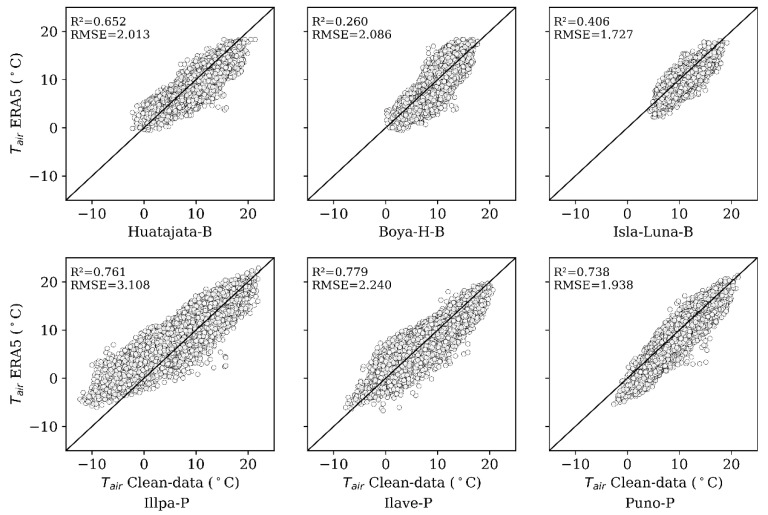
Pairwise comparisons between ERA5 reanalysis data and cleaned temperature observations for each station. Each subplot displays the relationship for one station, with corresponding coefficients of determination (R^2^) and Root Mean Square Errors (RMSE) shown within the plots.

The results of two spatial gap-filling models, LR-Spatial (Linear Regression Spatial), and RF-Spatial (Random Forest Spatial) are presented on Figure 3 and Table 4 using raw data and clean data. The predictive performance of clean data is quite high, particularly with the RF-Spatial model. Using clean data, the range of RMSE (Root Mean Square error) was 0.978 °C at Isla-Luna-B and 1.497 °C at Illpa-P, and RF-Spatial always beats LR-Spatial. At the Huatajata-B station, RF-Spatial achieved an $R^{2}$ of 0.923 as compared to 0.916 and RMSE 1.071 as compared to 1.122 in degrees Celsius. The minimum RMSE was 0.963 °C recorded at Isla-Luna-B with RF-Spatial. The beneficial feature of data preprocessing is obvious, since in most cases where raw data and clean data were compared, there was an undoubtedly higher level of correctness in the results obtained from the clean data. This shows that intensive quality control before modeling is warranted [70]. Moreover, at other stations such as Isla-Luna-B and Ilave-P, the RMSE decreases significantly (0.1–0.2 °C) between LR-Spatial and RF-Spatial with clean data, underlining the added value of machine learning in spatial air temperature ($T_{air}$) analysis.

#### 4.2.2. Temporal Gap-Filling Approaches on $T_{air}$

Temporal gap-filling of air temperature ($T_{air}$) was assessed based on the performance of different machine learning algorithms (Random Forest (RF), Support Vector Machine (SVM), AdaBoost (ADA), and a Stacked Ensemble Model (STACK)) alongside SVD-DE. These models were trained on raw data as well as quality-controlled data (clean data), and the data was divided into training and testing parts. The training set had a mean of 9.71 °C (SD: 3.43 °C, range: −2.81 °C to 21.37 °C) and the testing set had nearly the same statistics (mean: 9.70 °C, SD: 3.40 °C, range: −2.25 °C to 19.90 °C). The statistical similarity of the subsets provided a reliable evaluation of the model.

**Figure 4:**
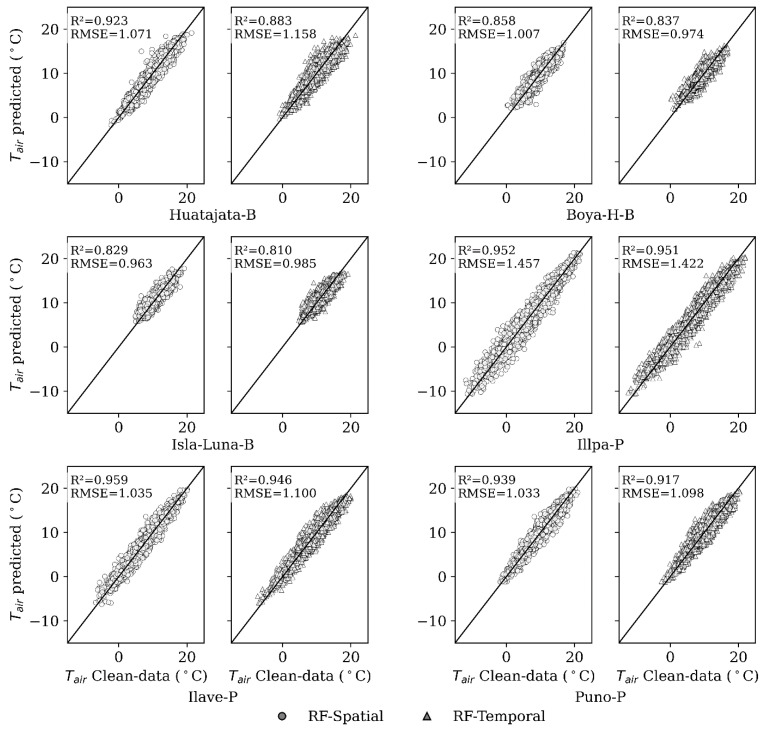
Comparison between observed and predicted $T_{air}$ generated by the spatial and temporal analysis, based on the clean-data dataset.

RF performed the best, or among the best overall among all the stations with the highest $R^{2}$ and the lowest RMSE in terms of training with clean data. The best performance was observed at the Illpa-P station, with $R^{2}$ of 0.951 and RMSE of 1.422 °C. In contrast, the lowest was observed at the Isla-Luna-B station, with $R^{2}$ of 0.810 and RMSE of 0.985 °C, see Figure 4. These findings remind us of the significance of preprocessing the data, since the accuracy of the models increased greatly after preprocessing. RF turned out to be the most reliable algorithm used in $T_{air}$ gap filling, especially when quality-controlled data were used to train it.

**Figure 5:**
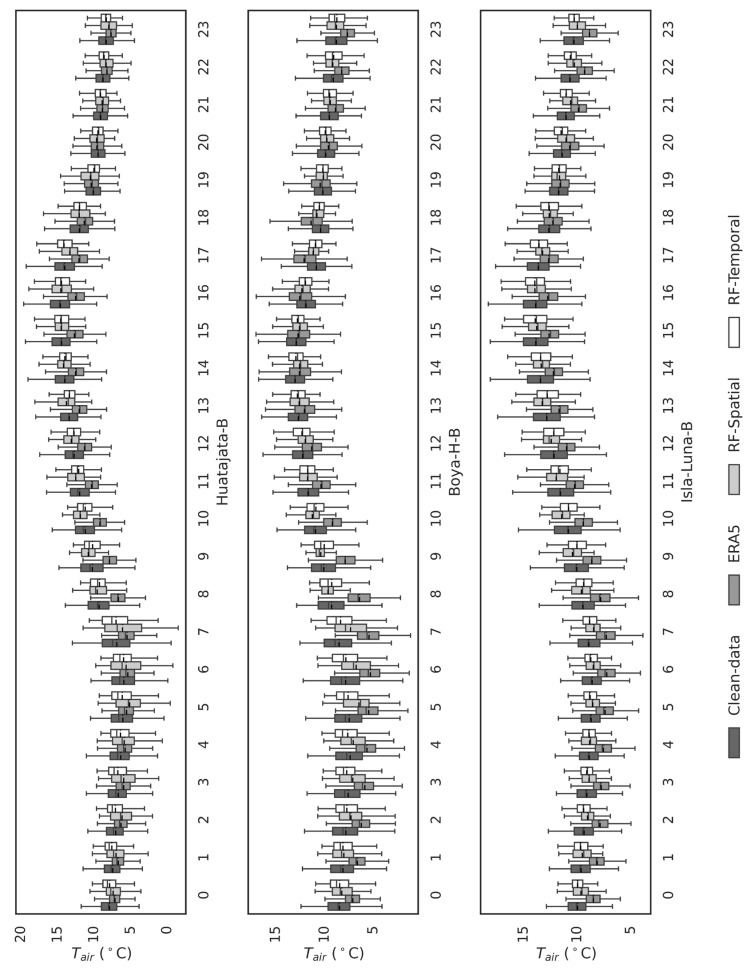
Boxplot comparison of observed and model-predicted temperature values (Temporal-Based and Spatial-Based models) for each hour of the day, using the clean data dataset for the first three stations.

**Figure 6:**
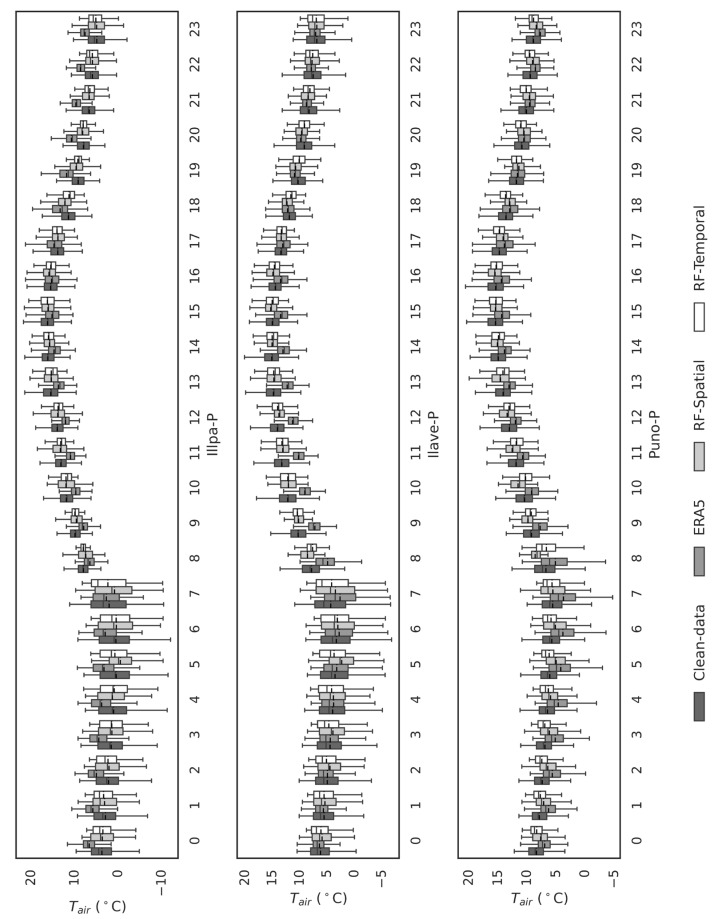
Boxplot comparison of observed and model-predicted temperature values (Temporal-Based and Spatial-Based models) for each hour of the day, using the clean data dataset for the last three stations.

Although ERA5 was an effective auxiliary dataset, it was not effective as a gap filler in the Lake Titicaca region at all. Figure 3 shows very low values of $R^{2}$ compared to clean data, which implies an insignificant agreement. It was found that ERA5 had systematic biases (over- or under-estimation) in some of the AWS stations, and performance varied spatially depending on the closeness to the lake, topography of the area, and elevation level. Also, Figure 5 and Figure 6 indicate that ERA5 has difficulty reconstructing the diurnal temperature patterns, especially in the early morning and late afternoon.

Finally, Table 5 provides the evaluation indicators of the machine learning models used by the Titicaca stations, with the ERA5-Land variables. In total, all tested models, including SVM, RF, ADA, and STACK, demonstrated a good level of accuracy in terms of the relatively low value of the RMSE and high values of $R^{2}$. Interestingly enough, the $R^{2}$ value of Random Forest was highest, meaning that it had a better ability to capture nonlinear dynamics of temperatures. AdaBoost was also good in terms of bias reduction, which was evident in their lower RMSE levels. The general improvement of performance to all models when the ERA5-Land variables are used testifies to the importance of performing multi-source data assimilation, further proving why it has become so essential in enhancing temperature modeling.

Table 6 offers the values of bias between the model types, ERA5, RF-Spatial, and RF-Temporal, compared to the clean data at various meteorological stations. ERA5 displays much greater values of bias (varying between −1.260 and 1.251), which points to the rates of over- and under-estimations between individual stations. In sharp contrast, the RF-Spatial and RF-Temporal machine learning models have much lower bias. The bias of RF-Spatial is between −0.064 and 0.047, whereas the RF-Temporal model’s range of bias is between −0.076 and 0.013. These findings are very indicative of the fact that Random Forest models perform better in terms of bias reduction compared to ERA5.

## 5. Discussion

The combined use of global (IQR, Biweight) and local (LOF) outlier detection methods improves sensitivity to different types of anomalies. Each method responds to distinct data characteristics. Global methods focus on overall climatological distributions, while local methods assess deviations relative to nearby observations.

Requiring a data point to be flagged by all three methods significantly reduced false positives and improved detection reliability; however, this criterion may also increase false negatives, meaning that real anomalies may be overlooked if they are not consistently detected by all algorithms. This conservative approach emphasizes robustness and confidence in the identified outliers but may miss extreme values that are only captured by a specific method. Nevertheless, given the objective of ensuring high data quality data for subsequent analyses, this strategy represents a deliberate choice that prioritizes detection reliability over the exhaustive identification of all possible anomalies [71]. This limitation is inherent to multi-method approaches and highlights the importance of using complementary evaluation metrics to assess detection performance more comprehensively.

Moreover, the gap-filling evaluation showed that the RF model achieved better results compared to the other tested algorithms, particularly when applied to clean data (see Table 5). These results highlight the importance of data quality, as both spatial and temporal gap-filling performance improved significantly after removing outliers and inconsistencies. In spatial RF based on inter-station correlations, a reduced dispersion was observed at stations like Huatajata-B, Isla-Luna-B, Ilave-P, and Puno-P, likely due to sthe trong correlations between neighboring stations (see Table 4). In contrast, the temporal RF model yielded better results for stations like Boya-H-B and Illpa-P where local meteorological variability and data scarcity limited the effectiveness of spatial interpolation.

Both gap-filling approaches, spatial (based on data from nearby stations) and temporal (based on the station’s own time series), showed high performances, with $R^{2}$ values consistently above 0.8 and RMSE close to 1 °C. This represents a substantial improvement over ERA5, which showed larger errors (~2 °C) and lower correlations, see Figure 3. The spatial approach generally yielded better results, with higher $R^{2}$ and lower RMSE compared to the temporal approach. For example, in Huatajata-B, $R^{2}$ increased to 0.923 and RMSE decreased from 1.158 to 1.071. In contrast, Illpa-P showed minimal variation between methods ($R^{2}$ from 0.951 to 0.952; RMSE from 1.422 to 1.457).

In the spatial approach, the homogeneity of the observed data and the availability of nearby stations are key factors. In contrast, the temporal approach leverages the extensive coverage of ERA5 data to provide stability in areas with sparse station networks. Both methods show good statistical performance for gap filling. Figure 4 shows the comparison of $T_{air}$ estimates from two Random Forest models: (a) spatial (RF-Spatial); and (b) temporal (RF-Temporal). These results support the use of a hybrid approach, applying spatial models where nearby stations show strong correlations, and temporal models where such correlations are weak or station data are limited.

Figure 5 presents annual hourly boxplots (0–23 h) comparing observed temperatures (clean data) with predictions from spatial and temporal models for each AWS. The models, particularly Random Forest (RF), accurately replicate hourly variations, including medians and interquartile ranges, effectively capturing the diurnal variability critical for representing extreme events. Discrepancies are more pronounced between 00:00 and 11:00 (likely due to thermal inversions or lack of solar radiation) and 18:00–23:00 (with higher sensitivity in land-based stations). Both spatial and temporal models significantly outperform ERA5, which exhibits substantial biases and poor adaptation to local hourly variability (see also Figure 6). These discrepancies are likely due to ERA5′s inability to account for fine-scale atmospheric processes and local environmental factors, which are crucial in accurately capturing temperature fluctuations at these times. Figure 5 and Figure 6 provide a detailed side-by-side comparison of the observed and predicted $T_{air}$ through hourly boxplots for all stations (Huatajata-B, Boya-B, Isla-Luna-B, Illpa-P, Ilave-P, and Puno-P). The predicted distributions closely match the observed data (clean data), with similar interquartile ranges and median values across most hours. The RF-based spatial analysis excels at replicating the diurnal cycle, including amplitude, maxima, and minima. In contrast, ERA5 fails to reproduce observed distributions, particularly during early morning (00:00–11:00) and late afternoon/evening (18:00–23:00), underscoring the need for complementary tools or ERA5 data refinement.

The comparison between the observed data and ERA5 reveals significant biases, see Table 6, particularly in capturing temperature variation. In contrast, machine learning models effectively address these biases by integrating high-resolution ERA5-Land data with ground-based observations. This approach results in improved predictions that closely align with the observed temperature distributions (Figure 5 and Figure 6), highlighting the advantage of machine learning models in reducing the discrepancies inherent in ERA5, particularly in data-scarce regions.

Shapiro–Wilk normality tests and visual analysis (Figure 5 and Figure 6) reveal ERA5′s inability to capture the true distribution shape, displaying biased patterns at specific hours: Huatajata-B: Hours 9, 13–14, 18–20, and 22:00, Boya-H-B: 9:00–21:00, Isla-Luna-B: 7:00–17:00, Illpa-P: 15:00–20:00, Ilave-P: 9:00–17:00, Puno-P: 12:00–18:00. The lower bias in the machine learning models (Table 6), can be attributed to their ability to capture local patterns and temporal trends that ERA5 fails to grasp. These biases highlight ERA5′s limitations in representing local hourly dynamics, reinforcing the superiority of the developed models.

The temperature estimation models (especially RF) demonstrate high sensitivity to input data quality, with notable improvements after rigorous quality control. The close alignment between the predicted and observed distributions, both visually and statistically, validates the success of the data-cleaning and modeling process. The results advocate for refined approaches over direct ERA5-Land use, as the models provide more realistic predictions with representative distributions.

## 6. Conclusions

A comprehensive procedure for the quality control and gap filling of hourly temperature data from six automatic weather stations (AWS) in the Lake Titicaca region is described and applied to observations spanning from 2019 to 2022. The quality control phase identified statistical outliers using the Interquartile Range, Biweight, and Local Outlier Factor methods. In addition, range, step, and persistency tests were applied to detect further inconsistencies. The quality control process resulted in the removal of 0.005% (Boya-H-B) to 17.56% (Illpa-P) of the raw data, yielding a clean dataset.

Gap-filling with a temporal approach was carried out using four machine learning algorithms (Random Forest, Support Vector Machine, Stacking, and AdaBoost, with 70% of the ground-based hourly temperature observations and ERA5-Land data used for the training. Random Forest was the most efficient algorithm, with validation metrics showing bias between −0.076 and 0.013 °C, RMSE between 0.98 and 1.45 °C, and R^2^ between 0.81 and 0.95. In the spatial approach, RF also outperformed the linear correlation regression based on nearby stations, with the bias of that model being between −0.03 and 0.047 °C, RMSE between 0.96 and 1.5 °C, and R^2^ between 0.81 and 0.96. The spatial approach was more effective in networks with strong interstation correlations, while the temporal approach performed better for stations with weak correlations and higher local variability. These results suggest a selective strategy could be effective: applying spatial models where nearby stations are correlated, and a temporal approach where such references are lacking.

## Figures and Tables

**Table 1:** Geographic coordinates and instrumentation details.

Stations	Longitud	Latitud	Altitud (m.a.s.l.)	Technical Description
Isla de Luna, Bolivia (Isla-Luna-B)	69° 03′ 44.28″ W	16° 02′ 50.92″ S	3812	Campbell Scientific CSRAWS, Logan (Utah), USA. Equipped with rain gauge (TE525WS-L) [31], probe for RH/T (CS215) [32], anemometer (A100R) [33], wind vane (W200P) [33], pyranometer (SP1100) [34].
Huatajata, Bolivia (Huatajata-B)	68° 41′ 50.81″ W	16° 12′ 41.09″ S	3831	Campbell Scientific CSRAWS, Logan (Utah), USA. Equipped with rain gauge (TE525WS-L) [31], probe for RH/T (CS215) [32], anemometer (A100R) [33], wind vane (W200P) [33], pyranometer (SP1100) [34].
Boya-HidroMet, Bolivia (Boya-H-B)	68° 40′ 26.76″ W	16° 14′ 23.28″ S	3811	Vaisala WXT520, Vantaa, Finland [Vaisala, 2019]. Equipped with rain gauge (OTT Pluvio2) [35], sensors for RH/dew point/air temperature (HMP110, HMP155) [36], anemometers (WA15, WMT52, WMT703) [37], pyranometer (Li-200R) [38].
Puno, Perú (Puno-P)	70° 00′ 43.56″ W	15° 49′ 34.68″ S	3820	Vaisala WXT520, Vantaa, Finland [Vaisala, 2019]. Equipped with rain gauge (OTT Pluvio2) [35], sensors for RH/dew point/air temperature (HMP110, HMP155) [36], anemometers (WA15, WMT52, WMT703) [37]
Illpa, Perú (Illpa-P)	70° 04′ 47.28″ W	15° 40′ 51.24″ S	3827	Vaisala WXT520, Vantaa, Finland [Vaisala, 2019]. Equipped with rain gauge (OTT Pluvio2) [35], sensors for RH/dew point/air temperature (HMP110, HMP155) [36], anemometers (WA15, WMT52, WMT703) [37]
Ilave, Perú (Ilave-P)	69° 37′ 33.24″ W	16° 05′ 17.52″ S	3837	Vaisala WXT520, Vantaa, Finland [Vaisala, 2019]. Equipped with rain gauge (OTT Pluvio2) [35], sensors for RH/dew point/air temperature (HMP110, HMP155) [36], anemometers (WA15, WMT52, WMT703) [37]

**Table 2:** Summary of outliers and erroneous values from integral analysis.

		Weather Monitoring Network					
Description		Huatajata-B	Boya-H-B	Isla-luna-B	Puno-P	Illpa-P	Ilave-P
Record period		2019-09-13T00 2022-04-07T13	2019-09-13T00 2022-04-07T13	2019-09-13T00 2022-04-07T13	2019-09-13T00 2022-04-07T13	2020-05-01T00 2022-04-07T13	2020-05-01T00 2022-04-07T13
Gaps, %		0.00	7.73	0.00	0.30	5.87	0.09
raw data records		22,502	20,763	22,502	22,435	15,962	16,942
Outlier values from raw data		143 (0.77%)	200 (0.96%)	265 (1.18%)	229 (1.02%)	156 (0.98%)	190 (1.12%)
Consistency tests *							
Range test	Tlow < Tair < Thigh	0	0	0	0	0	10
Persistency test	ThTh-1Th-2Th-3	0	0	0	4	7	3
Step test	|Th-Th-1| < 4	171	1	3	210	953	359
	|Th-Th-2| < 7	20	0	0	35	1109	392
	|Th-Th-3| < 9	4	0	0	20	1291	543
	|Th-Th-6| < 15	0	0	0	7	1046	244
	|Th-Th-12| < 25	0	0	0	0	25	0
Erroneous values from consistency tests		187 (0.831%)	001 (0.005%)	003 (0.013%)	252 (1.123%)	2803 (17.560%)	1098 (6.481%)
Overlapping values with outliers		9	--	--	18	32	20
clean data		22,142	20,562	22,234	21,944	13,003	15,654

* The values obtained from the persistency test and the step test were not considered for the time serial clean data.

**Table 3:** $R^{2}$ and RSME values for all time series of $T_{air}$ raw data.

	Huatajata-B	Boya-H-B	Isla-Luna-B	Illpa-P	Ilave-P	Puno-P
Boya-H-B	0.89/1.70					
Isla-Luna-B	0.84/2.23	0.79/1.89				
Illpa-P	0.90/3.76	0.85/4.65	0.74/5.49			
Ilave-P	0.91/2.20	0.87/2.94	0.79/3.66	0.96/2.31		
Puno-P	0.88/1.83	0.81/2.35	0.84/2.36	0.89/3.68	0.92/2.06	0.92/2.06

**Table 4:** $R^{2}$ and RMSE values when using LR and RF models at spatial scale.

	Huatajata-B		Boya-H-B		Isla-Luna-B		Illpa-P		Ilave-P		Puno-P	
	Raw-data	Clean-data	Raw-data	Clean-data	Raw-data	Clean-data	Raw-data	Clean-data	Raw-data	Clean-data	Raw-data	Clean-data
$R^{2}$/												
LR-Spatial	0.901	0.916	0.825	0.840	0.792	0.818	0.939	0.950	0.952	0.957	0.892	0.933
RF-Spatial	0.913	0.923	0.844	0.858	0.815	0.829	0.945	0.952	0.956	0.959	0.911	0.939
RMSE/												
LR-Spatial	1.142	1.122	1.071	1.069	1.035	0.991	1.581	1.488	1.077	1.059	1.335	1.084
RF-Spatial	1.065	1.071	1.011	1.007	0.978	0.963	1.497	1.457	1.034	1.035	1.217	1.033

**Table 5:** Comparison of (a) R^2^ and (b) RMSE performance metrics for Random Forest (RF), Support Vector Machine (SVM), AdaBoost (ADAB), and Stacking (STACK-RF) models across different model scenarios on the testing dataset.

	Huatajata-B		Boya-H-B		Isla-Luna-B		Illpa-P		Ilave-P		Puno-P	
	Raw data	Clean data	Raw data	Clean data	Raw data	Clean data	Raw data	Clean data	Raw data	Clean data	Raw data	Clean data
$R^{2}$												
RF	0.876	0.883	0.830	0.837	0.793	0.810	0.944	0.951	0.938	0.946	0.908	0.917
SVM	0.794	0.808	0.755	0.762	0.741	0.753	0.887	0.891	0.883	0.895	0.860	0.867
ADAB	0.807	0.830	0.736	0.757	0.702	0.735	0.898	0.920	0.883	0.907	0.846	0.876
STACK	0.874	0.881	0.828	0.835	0.791	0.806	0.943	0.949	0.937	0.945	0.906	0.915
RMSE												
RF	1.197	1.158	1.001	0.974	1.040	0.985	1.446	1.422	1.173	1.100	1.155	1.098
SVM	1.540	1.485	1.200	1.178	1.164	1.124	2.056	2.113	1.605	1.536	1.422	1.385
ADAB	1.491	1.396	1.247	1.189	1.249	1.165	1.952	1.810	1.607	1.452	1.492	1.336
STACK	1.207	1.169	1.005	0.979	1.046	0.995	1.464	1.441	1.182	1.111	1.169	1.105

**Table 6:** Bias values, compared to the clean data across several meteorological stations.

Clean Data	Huatajata-B	Boya-H-B	Isla-Luna-B	Illpa-P	Ilave-P	Puno-P
ERA5	−1.021	−0.998	−1.224	1.251	−0.678	−1.260
RF-Spatial	−0.033	−0.030	0.012	−0.006	0.047	−0.064
RF-Temporal	−0.076	−0.046	0.013	−0.054	−0.032	0.005

## Data Availability

Data are contained within the article.
